# Relational psychiatry and suicidal states: reclaiming hope in everyday clinical practice

**DOI:** 10.1192/bji.2025.10066

**Published:** 2025-11

**Authors:** Subodh Dave, Jon van Niekerk, Philip Pirie, Jo O’Reilly

**Affiliations:** 1 Consultant Liaison Psychiatrist, Derbyshire Healthcare NHS Foundation Trust, Derby, UK. Email: subodhdave@nhs.net; 2 Group Medical Director, Clinical Directorate, Cygnet Health Care, London, UK; 3 Independent Scholar, UK; 4 Consultant Medical Psychotherapist, Camden and Islington Mental Health and Social Care Trust, London, UK

**Keywords:** Risk assessment, suicide, medical leadership, biopsychosocial formulation, safety planning

## Abstract

The growing demand for psychiatric services, coupled with the increasing complexity of clinical presentations, is compounded by systemic pressures – among them inadequate resources, fragmented service configurations, and regulatory and legal frameworks that seem to apportion blame to the individual rather than recognising the wider systemic context. These factors can leave clinicians feeling disempowered and demoralised. This editorial is a call to renew hope, to reaffirm that psychiatrists, using their everyday medical and psychiatric expertise in personalising the biopsychosocial care they provide to their patients, can make a critical difference when dealing with suicidal states. Effective relational psychiatry offers hope to both clinicians and patients. We must not lose it.

At a time of growing complexity and pressure within mental health systems globally, expectations of suicide prevention can feel like an overwhelming task. Clinicians may feel powerless, fatigued and demoralised by systems that appear to offer too little, too late. Against this backdrop, therapeutic nihilism, the quiet erosion of belief that our interventions matter or the complexity and dilemmas inherent within our work will be acknowledged, has become a significant and under-recognised risk to safe and effective care.

This editorial is both a reaffirmation of psychiatry’s central role in working with suicidal states of mind and a call to renew hope. Drawing on NHS England’s *Staying Safe from Suicide* guidance,^1^ recent data and clinical experience, we argue that psychiatrists remain uniquely placed to reduce suicide risk, not through grand interventions or predictive tools, but through presence, formulation and good clinical medicine. We write with humility, recognising the limits of our influence, but also with conviction that psychiatry, when practised relationally, has the potential to relieve intense distress and suicidal despair, thereby reducing suicidal risk.

## The global challenge of suicide prevention

Each year, more than 700 000 people die by suicide worldwide, with many more making attempts.^2^ In England and Wales alone, over 6000 deaths were registered in 2023, an average of 19 lives lost each day.^3^ Tragically, five of these daily deaths involved individuals under the care of mental health services, and four of those five had been assessed as ‘low risk’.^4^ These sobering statistics have contributed to growing clinician disillusionment, particularly regarding the predictive value of traditional risk assessment tools.

## From risk scores to relational safety

The NHS England *Staying Safe from Suicide* guidance^1^ rejects the use of categorical risk stratification and numerical scoring, citing their poor predictive validity and the dangers of false reassurance. This builds on long-standing guidance from the National Institute for Health and Care Excellence (NICE),^5^ which cautions against the use of risk prediction tools to determine access to services or interventions, given their limited utility in accurately predicting suicide. Instead, it advocates a paradigm shift: one that centres relational practice, collaborative formulation and safety planning.

This approach, framed through the triad of safety assessment, safety formulation and safety planning, offers a clinically credible alternative to checklist-based assessments. It restores narrative understanding and recognises that safety emerges not from categorisation, but from connection. In this respect, it represents a paradigm shift for psychiatrists and patients, modelling collaboration, a shared and agreed safety plan, timely and urgent interventions when needed and realistic thinking beyond despair and anguish towards recovery. The message is simple yet profound: good psychiatry is protective – not in isolation or through perfect foresight, but through presence, curiosity, continuity and compassion.

## Reclaiming the fundamentals of clinical practice

Hope can be found in the fundamentals. Curiosity and the capacity to be alongside and contain intense emotional distress. Offering therapeutic interventions based on an understanding of the person behind the symptoms and what matters most to them. Revisiting diagnosis. Exploring treatment resistance and all the factors that may contribute to this through a broad lens beyond the pharmacological. Asking whether trauma has been explored or distress has been misread. Reviewing medication, dosage, duration, interactions and overlooked dual diagnoses. Supporting the workforce with the emotional impact of their work so they too feel contained and are able to think under pressure. These are not acts of defensive practice; they are expressions of clinical care, of good psychiatric practice and of effective medical practice.

Clearly, public health-based primary prevention measures have an important role to play in suicide prevention.^6^ However, without compassionate and open-hearted clinical practice these measures will not deliver the results we seek. Targeted secondary prevention through early intervention, for example in young-onset psychosis, has been shown to reduce suicides and suicidal behaviour.^7^ Given the fortunate rarity of suicide, it is difficult to demonstrate the suicide-mitigating impact of good mental healthcare. That should not negate the safety impact of the bread and butter work we psychiatrists do, in managing severe mental illness, with its common comorbidities such as alcohol misuse.

This is especially important in low- and middle-income countries, where resources are scarce, workforce shortages acute and suicide rates often high. Relational psychiatry costs little, yet can offer much. In these contexts, the psychiatrist’s ability to build therapeutic alliances, co-produce meaning, and work with families becomes even more central. Quaternary prevention, protecting patients from harm caused by over-medicalisation or institutional risk aversion, is also a global imperative. The pressure to manage risk with rigid protocols can erode relational safety and foster disengagement. We must resist the urge to replace clinical judgement with procedural compliance.

## Safety planning: a small, but powerful tool

One of the most hopeful interventions in suicide prevention is also one of the simplest: the safety plan. Co-produced with the person seeking treatment, a safety plan includes warning signs, internal coping strategies, people to contact and steps to make the environment safer^8^ based on the individual’s unique circumstances. In England, *Staying Safe from Suicide* makes safety planning mandatory in both in-patient and community settings,^1^ and organisations such as Staying Safe (StayingSafe.net) offer accessible, co-produced tools and templates that are now in use across services.

Safety planning is not just a document, it offers a containing clinical relationship that helps the person feel understood and empowered. It fosters therapeutic trust and provides structure in times of emotional dysregulation. Its core message is one of hope: ‘you matter, and we will plan together for when things feel overwhelming’.

## Beyond the system: the clinician as a source of hope

Systemic pressures are real; burnout, moral injury, fractured services with rising demand seem to be the norm. But even within these constraints, clinicians can make a difference. A moment of curiosity, a shared formulation, a revisited diagnosis, a co-created plan – these are modest acts that carry disproportionate impact.

The relational psychiatrist does not need to be beholden to the impossible task of trying to predict suicide. Instead, we can walk alongside distress, invite meaning and offer containment when hope feels lost. Central to this is our own professional recognition that staff can best offer this to patients when they are supported and contained within our places of work, are able to share dilemmas and anxiety inherent in the work and to learn from each other; relationships lie at the heart of care for both clinician and patient. At best, a psychiatrist’s work represents a deeply human act of compassion and containment, and one that aligns with the therapeutic heart of psychiatry.

Hope, then, is not naive optimism. It is a belief (grounded in evidence and experience) that connection can transform. It is the clinician’s refusal to give up, even when systems falter.

## Operationalising relational safety in practice

For suicide prevention to move from policy to practice, relational practice must be operationalised at every level of care. This requires active engagement with a wide spectrum of stakeholders: patients and carers, multidisciplinary teams, professional regulators, policymakers and the legal frameworks that scrutinise care following a suicide. Collaborative approaches are essential: engaging lived experience networks to co-design safety planning tools; involving front-line clinicians in embedding safety formulation within routine practice; and ensuring managers and commissioners understand the time, supervision and continuity relational work requires. In many jurisdictions, coronial or other processes following a suicide can shape public perception, leading to unrealistic expectations of staff and their professional accountability. This makes it all the more critical that documentation reflects meaningful engagement and sound clinical reasoning, rather than mere procedural compliance. Internationally, this means working with oversight bodies and quality regulators to advocate for frameworks that value therapeutic connection alongside other measurable outcomes.

## The way forward

As psychiatrists, we have both the privilege and responsibility to hold this hope, for our patients and ourselves. The Staying Safe framework offers an opportunity to do just that. But its success depends on more than training or policy; it relies on our willingness to practise relationally, to reflect honestly and to remain curious about those we care for.

In doing so, we reaffirm that psychiatry, at its best, is not just about preventing death. It is about dignifying life.

## References

[1] NHS England. Staying *Safe from Suicide*. NHS England, 2025 (https://www.england.nhs.uk/long-read/staying-safe-from-suicide/ [accessed 4 Aug 2025]).

[2] World Health Organization. Suicide Worldwide in 2023: Global Health Estimates. WHO, 2023.

[3] Office for National Statistics. *Suicides in England and Wales: 2023 Registrations*. ONS, 2024 (https://www.ons.gov.uk/ [accessed 4 Aug 2025]).

[4] National Confidential Inquiry into Suicide and Safety in Mental Health. *Annual Report 2023: UK Patient and General Population Data 2010–2020*. University of Manchester, 2023 (https://sites.manchester.ac.uk/ncish/reports/annual-report-2023/).

[5] National Institute for Clinical Excellence. Self-Harm: Assessment, Management and Preventing Recurrence (NICE Guideline NG225). NICE, 2022 (https://www.nice.org.uk/guidance/ng225). 36595613

[6] Pirkis J , Dandona R , Silverman M , Khan M , Hawton K . Preventing suicide: a public health approach to a global problem. Lancet Public Health 2024; 9: e787–95. 39265611 10.1016/S2468-2667(24)00149-X

[7] Tahmazov E , Bosse J , Glemain B , Chiou MT , Chiu HJ , Langford PR , et al. Impact of early intervention for early psychosis on suicidal behavior – a meta-analysis. Acta Psychiatr Scand 2025; 151: 127–41. 39601245 10.1111/acps.13773 PMC11695091

[8] Stanley B , Brown GK. Safety planning intervention: a brief intervention to mitigate suicide risk. Cogn Behav Pract 2012; 19: 256–64.

